# In Situ Efficient End Functionalization of Polyisoprene by Epoxide Compounds via Neodymium-Mediated Coordinative Chain Transfer Polymerization

**DOI:** 10.3390/polym16182672

**Published:** 2024-09-22

**Authors:** Xiuhui Zhang, Jing Dong, Feng Wang, Xuequan Zhang, Heng Liu

**Affiliations:** 1 Shandong Provincial College Laboratory of Rubber Material and Engineering/Key Laboratory of Rubber-Plastics, Ministry of Education, School of Polymer Science and Engineering, Qingdao University of Science & Technology, Qingdao 266042, China; b023021001@mails.qust.edu.cn (X.Z.); fengwang@qust.edu.cn (F.W.); xqzhang@qust.edu.cn (X.Z.); 2 Petrochemical Research Institute, PetroChina, Beijing 102206, China; dongjing2@petrochina.com.cn

**Keywords:** neodymium, polyisoprene, functionalization, coordinative chain transfer polymerization

## Abstract

The Nd-mediated coordinative chain transfer polymerization (CCTP) of dienes represents one of the state-of-the-art techniques in the current synthetic rubber field. Besides having well-controlled polymerization behaviors as well as high atom economies, it also allows for the generation of highly reactive Al-capped polydienyl chain-ends, which hold great potential, yet much less explored up to date, in achieving end functionalization to mimic the structure of natural rubber. In this study, we demonstrate an efficient in situ method to realize end-functionalizing polyisoprene by introducing epoxide compounds into a CCTP system. The end functionalization efficiency was 92.7%, and the obtained polymers were systematically characterized by ^1^H NMR, ^1^H,^1^H-COSY NMR, DOSY NMR, and MALDI TOF. NMR studies revealed that a maximum of two EO units were introduced to the chain ends, and based on density functional theory (DFT) studies, an energy barrier of 33.3 kcal/mol was required to be overcome to open the ring of the EO monomer. Increasing the ratio of [Ip]/[Nd] resulted in gradually increased viscosities for the reaction medium and therefore gave rise to an end functionalization efficiency that decreased from 92.7% to 74.2%. The end hydroxyl group can also be readily converted to other functionalities, as confirmed by NMR spectroscopy.

## 1. Introduction

The neodymium-mediated coordinative chain transfer polymerization (CCTP) of conjugated diene monomers (e.g., 1,3-butadiene (BD), isoprene (Ip)) represents a highly efficient, highly atom-economic, and well-controlled strategy in the current synthetic rubber field [1,2,3,4,5,6,7,8,9,10,11]. In such a system, polydienyl propagating chains can undergo rapid and reversible chain transfer between Nd-based precatalysts and chain transfer agents (CTAs), which are usually found in the form of alkylaluminium compounds, revealing many advantages that cannot be achieved in traditional Nd-based catalytic systems. These advantages include the following: (1) Chain transfer occurs more rapidly than chain propagations, and diene monomers appear to be “synchronously” enchained in CTAs, which eventually results in precisely controlled molecular weights and extremely narrow molecular weight distributions, paralleling classical anionic living polymerizations. (2) Every CTA can be regarded as an initiator, enabling a single Nd-based precatalyst molecule to generate multiple polydienyl chains, which can be easily controlled by adjusting CTA equivalents, thus achieving high atom economy—a critical factor given the high cost of Nd compounds compared to other metals; this atom economic characteristic shows a contrast to classical anionic living polymerizations wherein one lithium catalyst can only generate one polymer chain. (3) During the end of CCTP, most of the polymer chains are end-capped with a CTA metal, i.e., most chains feature a C-Al bond in the chain end, which can undergo fast nucleophilic reactions with various heteroatom-based compounds, hence demonstrating an efficient end functionalization methodology. Because of these unique features, the CCTP of dienes has witnessed tremendous growth during the past few years [1,10,12,13,14,15,16,17,18,19,20,21,22,23,24,25,26,27,28].

Nd-based catalysts, among those used in industry for diene polymerization, exhibit the highest cis-1,4-selectivity, producing synthetic rubbers like NdBR and NdIR with a cis-1,4 content of up to 99%. These materials offer excellent elastomeric properties, high wear resistance, and good flex fatigue properties. Nevertheless, their overall mechanical properties still underperform natural rubber (NR) [4,29,30,31,32,33,34]. One main reason is that NdIR and NdBR lack functional groups, which results in the absence of functionality-accompanied strain-induced crystallization and inferior compatibility with polar inorganic fillers. Based on this consideration, the functionalization of NdIR and NdBR has been pursued by scientists for decades [4,29,30,32,34,35,36,37,38].

Despite the great advances in CCTP chemistry, as mentioned above, very few reports focus on the end functionalization of polydienes despite the high reactivities of Al-capped chain ends, showing a striking contrast to the polyolefin field [39,40,41,42,43,44,45]. Many studies on the CCTP of dienes are mainly focused on the preparation of amphipathic block copolymers [8,9,46,47,48], such as PBD-*b*-PCL, rather than end functionalization. A very representative chain-end functionalization example was disclosed by M. Visseaux in 2019 [49] in which benzophenone was employed to end-functionalize *trans*-1,4-polydienes to afford−CPh_2_OH-capped polymers. Inspired by the reactivities of aluminum alkyl compounds for the ring opening polymerization of epoxides [46], herein, we incorporate epoxide compounds to Nd-mediated CCTP systems in situ to put forward a highly efficient manner to introduce hydroxyl-OH end-functionalized polyisoprenes. The polyisoprenyl-Al chain ends revealed high reactivities to different types of epoxide compounds, giving end-functionalization efficiencies as high as 92.7%. The details of this research will be described below.

## 2. Materials and Methods

*Materials*: Unless otherwise noted, all chemical materials were purchased from commercial sources and used without further purification. All manipulations of air- and/or moisture-sensitive compounds were carried out under a dry and oxygen-free argon or nitrogen atmosphere using standard Schlenk techniques. The solvents (*n*-hexane and toluene) and isoprene were dried and distilled using CaH_2_ for 2 h and stored in ampoule bottles in a nitrogen atmosphere. Nd(vers)_3_ was obtained as a commercial product and diluted to 0.05 mol/L by hexane. The alkyl aluminum compounds DIBAH (Al(*i*-Bu)_2_H) and dichlorodimethylsilane (Me_2_SiCl_2_) were purchased from Akzo Nobel and diluted with hexane into 1 mol/L and 0.2 mol/L, respectively.

*General procedures for CCTP of isoprene*: All experiments were carried out in a dry nitrogen atmosphere. Quantitative Nd(vers)_3_, isoprene, DIBAH, and Me_2_SiCl_2_ were added to the ampoule using a syringe, and the ampoule was placed in a constant temperature water bath at 50 °C for 45 min to obtain a yellow-green homogeneous solution. The above prepared catalyst solution was added to 1.85 mol/L isoprene dissolved in hexane, and the mixed solution was reacted in a water bath at 50 °C for 4 h. Polymerizations were carried out for 4 h and eventually quenched by adding 2.0 mL acidified ethanol containing 2,6-di-*tert*-butyl-4-methylphenol as a stabilizer. The polymer was dried in a vacuum oven at 50 °C for 24 h to a constant weight.

### 2.1. General Procedures for End Functionalization of Isoprene

The polymerization steps are the same as described above; a quantitative epoxy compound was added to the ampoule bottle before the termination step of the above reaction, and the reaction was continued in a water bath at 50 °C for 4 h. 

### 2.2. Characterization

*Chemical structure and molecular weight:* The number of average molecular weights (*M*_n_) and number of molecular weight distributions (*M*_w_/*M*_n_) of polymers were measured using an Agilent PL-GPC 50 gel chromatograph at 40 °C, and tetrahydrofuran (THF) was used as eluent at a flow rate of 1.0 mL/min. The values of *M*_n_ and *M*_w_/*M*_n_ were calculated by using polystyrene calibration. Using a Virian Union-400 nuclear magnetic resonance instrument and CDCl_3_ as solvent (at room temperature), the microstructure was determined by ^1^H NMR, ^13^C NMR, and the ^1^H ^1^H-COSY spectra using the Virian Union-400 nuclear magnetic resonance apparatus in CDCl_3_ as solvent (at room temperature). DOSY NMR measurements were performed at 298 K on a Bruker Avance AQS600 NMR spectrometer operating at 400 MHz, where the gradient value was 2%~95%, the relaxation time was 3 s, and the diffusion time was 0.06 s. FTIR spectra were recorded on a Nicolet iS10 FTIR spectrometer produced by Thermo Company. MALDI-TOF MS analysis was performed on a Bruker’s Microflex LRF mass spectrometer using *trans*-2-[3-(4-*tert*-butylphenyl)-2-methylprop-2-enylidene]malonitrile (DCTB) as matrix and silver trifluoroacetate as dopants.

*Water contact angle measurements:* The measurement was performed with a Digiddrop contact angle meter (model: SDC-200s). The contact angle was measured with 0.5 μL of water on the surface of a slide coated with a polymer film (100 mg polymer/1 mL DCM), and the 3 s contact angle data were recorded. The result is the average of five runs for each experiment.

### 2.3. DFT Calculations

All of the density functional theory (DFT) calculations were performed with the Gaussian 09 program [50]. The geometry structures of all species were optimized without any constraints by using the B3LYP function including the empirical dispersion correction computed with Grimme’s D3 formula (B3LYP-D3), and the 6-31G * basis set was used for all of the atoms [51,52]. Vibrational frequency analysis calculations were also performed at the same level of theory to ensure that each optimized structure truly represents a minimum or a saddle point. The refined single-point energy calculations of optimized structures were carried out at the B3LYP-D3/6-311G ** level of theory, taking into account the solvation effect of toluene with the SMD solvation model [53].

## 3. Results and Discussion

**Figure 1:**
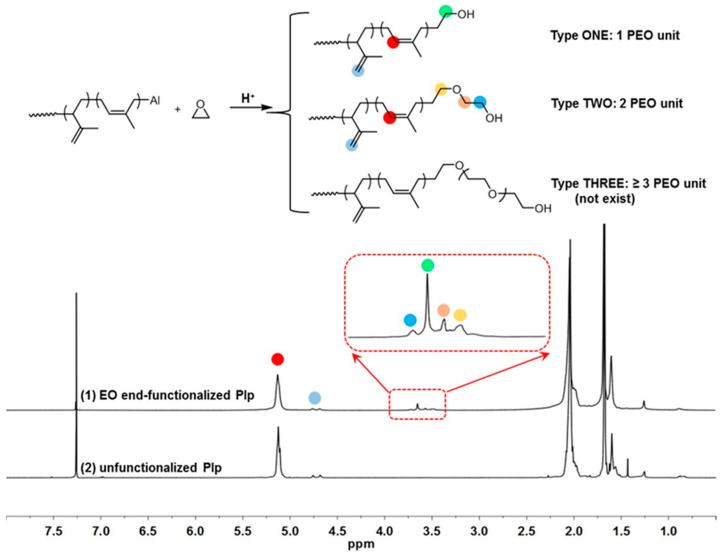
**Top**: Reaction scheme for end functionalization of PIP by EO; **bottom**: ^1^H NMR of (1) EO end-functionalized PIp and (2) unfunctionalized PIp.

**Figure 2:**
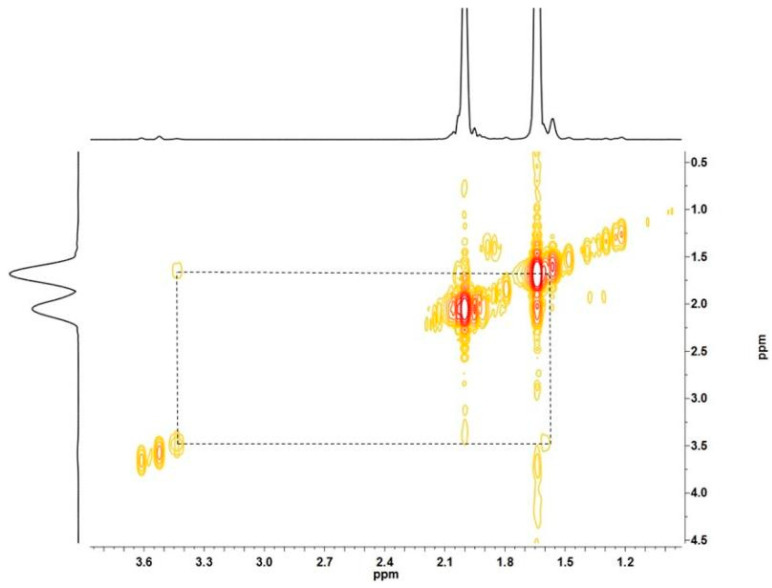
^1^H-^1^H COSY spectrum of EO end-functionalized PIp.

For Nd-mediated diene polymerization systems, diisobutylaluminum hydride (Al(*i*-Bu)_2_H, DIBAH) was found to be the most efficient CTA among all of the alkylaluminium compounds [6,8,9]. Therefore, the DIBAH-containing CCTP system of Nd(vers)_3_/Al(*i*-Bu)_2_H/Me_2_SiCl_2_ was intentionally selected herein to promote isoprene polymerizations. The end functionalization of such a system was next evaluated by using ethylene oxide (EO) as a representative epoxide compound. Moreover, in order to increase the Al-capped chain end concentration, which is beneficial to detect the expected hydroxyl chain ends after reacting with EO, polymerization was tentatively carried out at a relatively lower monomer ratio of [Ip]/[Nd] = 50 to decrease the molecular weights of the polyisoprene (PIp) product and a relatively higher ratio of [Al]/[Nd] = 30 to facilitate a chain transfer reaction (Table 1, entry 1). It was satisfying to find that, at a condition of [EO]/[Nd+Al] = 2, EO demonstrated very high reactivity with the polyisoprenyl-Al chain ends, affording the targeted PEO end-functionalized polyisoprenes in high yields. Figure 1 shows the ^1^H NMR spectra of the obtained polymer products from which the new resonance peaks at 2.94–3.78 ppm were assigned to methylene protons from -CH_2_O- units, verifying the successful enchainment of PEO into PIp chain ends. Moreover, it was found that, based on the different lengths of PEO units that originated from EO homopolymerizations, two types of PEO chain ends could be observed, including PIp with one PEO unit (Type ONE) and PIp with two PEO units (Type TWO). A PIp counterpart with ≥3 PEO units (Type THREE) was absent in the resultant product. For the former two end-functionalized PIps, Type ONE was predominant as it accounted for 83.3% of the total chain ends. Such two types of chain ends can be easily differentiated from the peaks located at 3.64 and 3.72 ppm, which were assigned to methylene protons in the terminal CH_2_OH group in Type ONE and Type TWO, respectively; the resonance peaks at 3.47 and 3.56 ppm were assigned to the in-chain methylene protons (-CH_2_O-) that were adjacent to oxygen atoms (Figure 1 (top)) in Type TWO chain ends. Such assignments could also be verified from the ^1^H-^1^H COSY spectrum in Figure 2.

**Figure 3:**
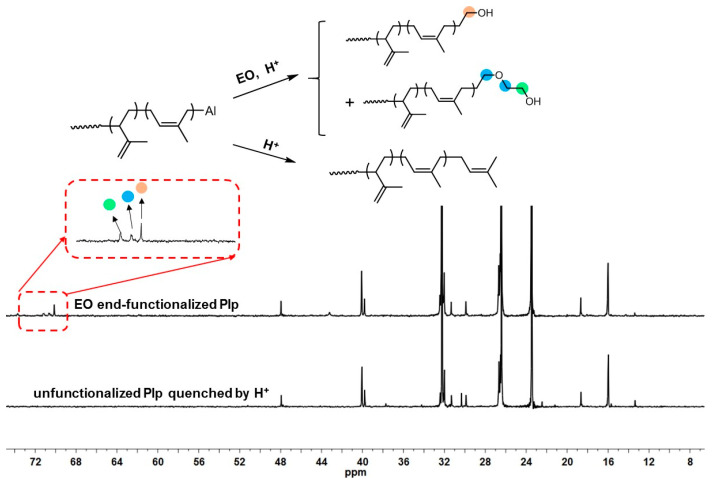
^13^C NMR of EO end-functionalized PIp and unfunctionalized PIp.

Based on the above ^1^H NMR analysis, the end functionalization efficiency (*f*) could be calculated using the following equation:

f=ICH2OH2×XnI=CH−+I=CH22

wherein *I_CH_*_2*OH*_ is the integrated area of methylene protons adjacent to the terminal hydroxyl group in the PEO units, *X_n_* is the average number of polyisoprene units per polymer chain, *I_=CH-_* is the integrated area of methine protons in the 1,4-polyisoprene unit, and *I_=CH2_* is the integrated area of methylene protons in the 3,4-polyisoprene unit. Based on such calculations, the end functionalization efficiency (*f*) was concluded to be 92.7%, indicating that most of the PIp chains were successfully end functionalized with polar PEO moieties. Such a high *f* value could also be confirmed from the ^13^C NMR spectra of the polymers (Figure 3) in which new resonance peaks at 71.16, 70.62, and 70.10 ppm that were assigned to the methylene carbons adjacent to oxygen atoms appeared.

**Figure 4:**
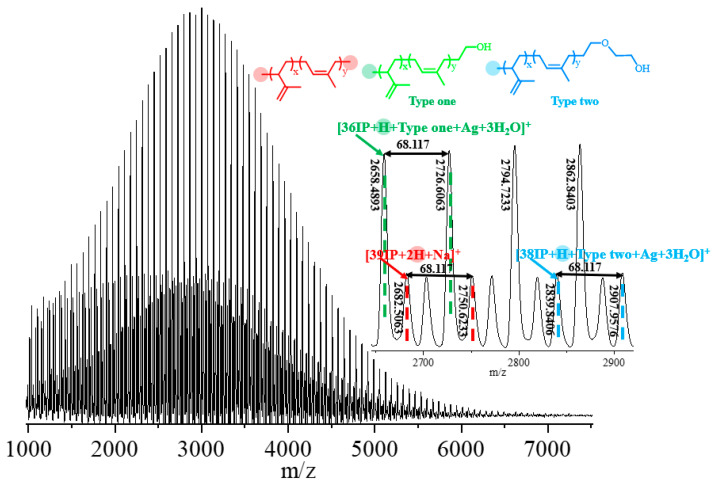
MALDI-TOF spectrum of EO end-functionalized PIp.

The prepared EO end-functionalized polyisoprene was characterized by an MALDI-TOF analysis. As shown in the spectrum in Figure 4, Type ONE can be clearly observed, with a small amount of Type TWO and being unfunctionalized PIp. Taking the peak of Type ONE (green) at 2727.2578 *m/z* as an example, it corresponded to 37 isoprene units (37 × 68.117 u) + the hydrogen of the α-end group (1.01 u) + the counterion Ag (106.91 u) + the Type ONE end group (45.0340 u) + 3 H_2_O in the system (3 × 18.0152 u). The small peak labeled with blue is the functionalized polyisoprene chain of Type TWO. The peak at 2839.4462 *m/z* corresponds to 38 isoprene units (38 × 68.117 u) + the hydrogen of the α-end group (1.01 u) + the counterion Ag (106.91 u) + the Type TWO end group (89.0603 u). The red-labeled small peak represents a small amount of unfunctionalized polyisoprene chain. The peak at *m/z* of 2681.6063 corresponds to 39 isoprene units (38 × 68.117 u) + the hydrogen of *α*- and *ω*-end groups (2 × 1.01 u) + the counterion Na (22.9898 u).

**Figure 5:**
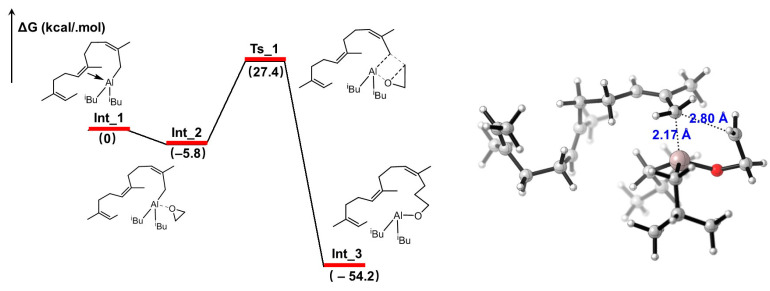
(**left**) Free energy profile of insertion step of EO into Al-capped polymer chain ends; (**right**) optimized structure for **Ts_1**.

The end-functionalization process of Al-capped polyisoprene by EO was also analyzed using density functional theory (DFT) studies. In such a study, intermediate **Int_1** bearing three polyisoprene units was established to model the Al-capped active species. It was found that, after the EO molecule coordinated to **Int_1** to give **Int_2** (Figure 5 (left)), an energy barrier of 33.3 kcal/mol was required to be overcome to open the ring of the EO monomer, and this process proceeded via a five-membered transition state of **Ts_1**, as shown in Figure 5 (right). At the end of the reaction, aluminum alkoxide species **Int_3** was generated, which could subsequently give rise to the corresponding hydroxyl group in the presence of acidic protons. Additionally, the overall insertion step was highly thermodynamically favorable, as concluded from the energy decrement of −54.2 kcal/mol, implying the high reactivity of EO with Al-capped polyisoprenyl chain ends, which also agreed well with the high end functionalization efficiency obtained above.

**Figure 6:**
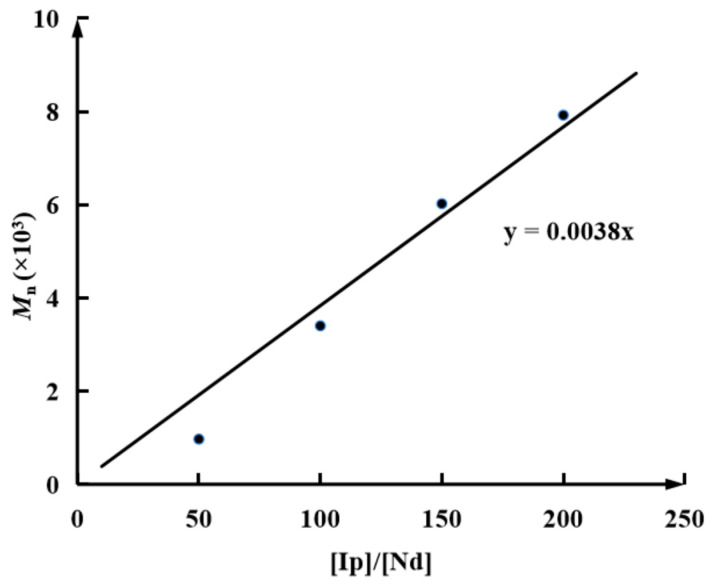
Plots of *M*_n_ of obtained PIps against [IP]/[Nd] ratios for EO end-functionalized CCTP system.

After establishing the basic reaction chemistry between the Al-capped polyisoprenyl chain and EO, studies were then expanded to polymerization at higher [Ip]/[Nd] ratios of 100, 150, and 200 wherein the concentration of Al-capped chain ends would be relatively lower. Nevertheless, it was found that such decreased concentrations of the active species posed a negative influence on their reactions with EO, giving gradually decreased end functionalization efficiencies ranging from 92.7% to 74.2%. When [Ip]/[Nd] ratios of 150 and 200 were applied to the system, the high viscosities of these reaction systems slowed down the reaction of the Al-capped chain ends with the EO molecule, giving very low functionalization efficiencies (Table 1, entries 3–4). Regarding the other parameters of the PIp products, their molecular weights showed a good linear relationship with the [Ip]/[Nd] ratios (Figure 6), and a gradually increased molecular weight was observed when increasing the [Ip]/[Nd] ratios, which was consistent with other CCTP systems. Such well-controlled behavior also manifested the superiority of end functionalization for a CCTP system versus other functionalization strategies, e.g., in-chain functionalization via a copolymerization manner wherein deactivated active species as well as ill-controlled performances are often observed [54,55,56].

**Figure 7:**
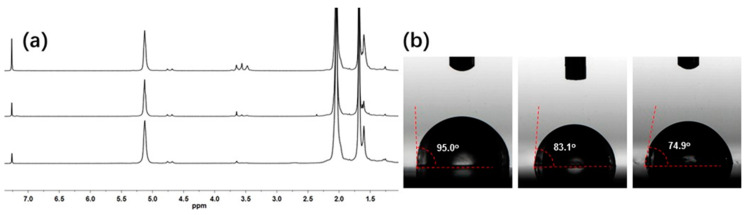
^1^H NMR (**a**) and WCA values (**b**) of EO end-functionalized PIp products.

**Figure 8:**
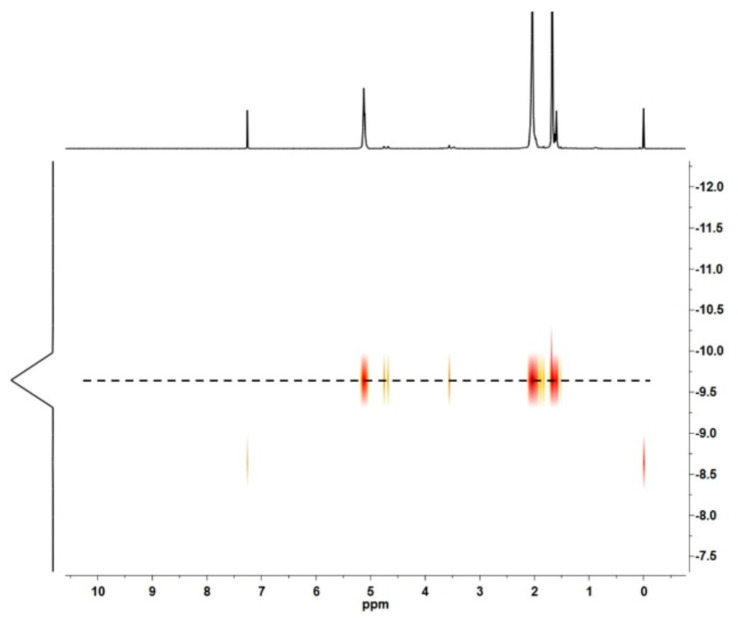
Two-dimensional DOSY NMR spectra of EO end-functionalized PIp.

The content of polar PEO ends could be varied by feeding more EO monomers into the CCTP system in situ. When increasing the [EO]/[Nd+Al] ratio from 2 to 4 and to 8, the intensities of the resonance peaks at 3.43–3.78 ppm, which were assigned to the -OCH_2_- protons, were clearly increased (Figure 7a), implying that the PEO contents gradually increased. A two-dimensional DOSY NMR experiment could exclude the formation of PEO homopolymers, because otherwise, two diffusion coefficients would be observed (Figure 8). Additionally, the increased polar PEO segments could obviously improve the surface properties of the resultant polymers, as revealed from the significantly decreased static water contact angles ranging from 95.0° to 74.9° (Figure 7b).

**Figure 9:**
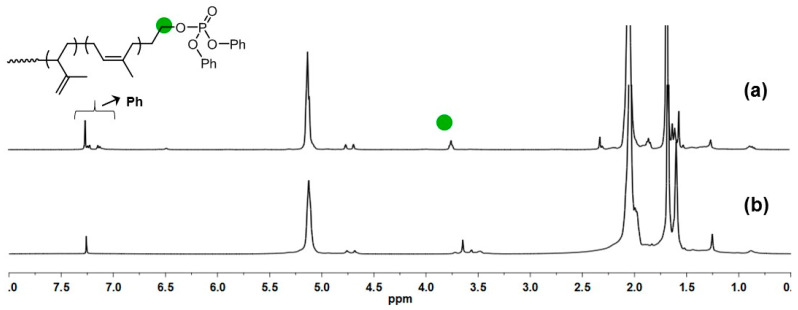
^1^H NMR spectra of DPCP-modified PIp (**a**) and pristine PEO-functionalized PIp (**b**).

The hydroxyl group in the PEO-functionalized PIp can be facilely converted to other functionalities due to its high catalytic activity. For instance, reacting hydroxyl groups with diphenyl chlorophosphite (DPCP) in the presence of triethyl amine could convert the CH_2_OH group into CH_2_OP(=O)(OPh)_2_ quantitatively, which could be obviously verified from the ^1^H-NMR spectrum where the methylene CH_2_ resonance peak shifted from the original 3.64 ppm to a lower field of 3.78 ppm (Figure 9).

## 4. Conclusions

In this study, the in situ end functionalization of polyisoprene, which was accessed from Nd-mediated coordinative chain transfer polymerization, by an epoxide compound is realized. Due to the high reactivity of the polyisoprenyl–Al bond that was generated during polymerization, the efficiency of end functionalization by epoxide can be as high as 92.7%, and the obtained functionalized polyisoprenes were well characterized by different methods, including NMR and MALDI-TOF, which also demonstrated that only 1–2 epoxide units were able to be incorporated to the chain ends, verifying the formation of end-functionalized polymers rather than block copolymers. Carrying out CCTP polymerization under high Ip/Nd ratios resulted in the end functionalization efficiencies gradually decreasing from 92.7% to 74.2%, which was perhaps due to the increased reaction viscosities.

## Figures and Tables

**Table 1:** End functionalization of PIp by EO by CCTP system of Nd(vers)_3_/Al(*i*-Bu)_2_H/Me_2_SiCl_2_^a^.

Run	[Ip]/[Nd]	Yield (%)	*M*_n_ ×10^3 b^	*M*_w_ ×10^3 b^	Đ ^b^	1,4-% ^c^	3,4-% ^c^	*f* ^d^
1	50	100	0.97	1.62	1.66	96.5	3.5	92.7
2	100	100	3.40	6.76	1.98	96.9	3.1	80.1
3	150	100	6.02	16.3	2.73	97.3	2.7	77.2
4	200	100	7.92	30.0	3.79	96.7	3.3	74.2

^a^ Polymerization conditions: [DIBAH]/[Nd] = 30, [Cl]/[Nd] = 3, *n*-hexane, 50 °C, 4 h, [EO]/[Nd+Al] = 2. ^b^ Determined by GPC using polystyrene standards, where Đ is dispersity index. ^c^ Determined by IR and NMR spectra. ^d^ *f* is end functionalization efficiency.

## Data Availability

The original contributions presented in the study are included in the article, further inquiries can be directed to the corresponding author.
